# Prevalence of Ectoparasitism on Small Ruminants in Kelantan, Malaysia

**DOI:** 10.21315/tlsr2020.31.1.3

**Published:** 2020-04-07

**Authors:** Vivi Susantie Syamsul, Ibrahim Abdul-Azeez Okene, Siti Nor Che Yahya, Ruhil Hayati Hamdan, Seng Hua Lee, Li Peng Tan

**Affiliations:** 1 Faculty of Veterinary Medicine, Universiti Malaysia Kelantan, Jalan Pengkalan Chepa, 16100, Kota Bharu, Kelantan, Malaysia; 2 Institute of Tropical Forestry and Forest Products, Universiti Putra Malaysia, 43400 UPM Serdang, Selangor, Malaysia

**Keywords:** Ectoparasites, Goats, Lice, Prevalence, Sheep, Ticks, Biri-biri, Ektoparasit, Kambing, Kutu, Prevalens, Sengkenit

## Abstract

Kelantan is a chiefly agrarian state with abundant small-holder ruminant farms in the East Coast economic Region of Malaysia. Ectoparasitism affects small ruminant production in Malaysia. It often causes reduction in meat quality and milk production which affect the farmers’ income. To date, no report for the prevalence of ectoparasitism on small ruminant in Kelantan compared to other state in Malaysia. This study aims to determine the prevalence and associated risk factor of ruminant ectoparasitism in Kelantan. Ectoparasites were collected by manual picking and skin scrapping from 462 sheep and goats in Kelantan between April and September 2017 (during dry season). 60% of the sampled animals were infested with at least one species of the ectoparasites. In this study, lice and ticks were the most prevalent ectoparasites on small ruminant, which were 43.64% and 22.98%, respectively. The high biotic potential of lice population on host might be one of the factors they become the most prevalent species found on the animals. There was no significant relationship between ectoparasitism prevalence and species of small ruminants (χ^2^ = 1.12, *p* = 0.293). However, there was significant variations in prevalence between the regions where the animals were sampled from (χ^2^ = 30.25, *p* = 0.002) and farm management system for both species. This present study provides baseline epidemiological data on the prevalence of ectoparasitism in small ruminant. This information is useful for the formulation of prevention and control measures in order to enhance ruminant productivity in Kelantan.

## Abstract (ms)

Kelantan adalah negeri agraria di kawasan ekonomi Pantai Timur Malaysia dengan kebanyakan penternak mempunyai ladang ruminan yang kecil. Ektoparasit menjejaskan pengeluaran ruminan kecil di Malaysia. Ia menyebabkan penghasilan kualiti daging yang rendah dan kurang penghasilan susu yang menjejaskan sumber pendapatan penternak. Buat masa ini, tiada laporan mengenai prevalens ektoparasitisme pada ruminan kecil di Kelantan jika dibandingkan dengan negeri-negeri lain di Malaysia. Kajian ini bertujuan untuk menentukan prevalens dan kaitan faktor risiko ektoparasitisme pada ruminan di Kelantan. Ektoparasit dikutip secara manual dan kikisan kulit daripada 462 ekor biri-biri dan kambing di Kelantan diambil di antara bulan April dan September (sepanjang musim kering). Sebanyak 60% daripada haiwan persampelan dijangkiti oleh sekurang-kurangnya satu spesis ektoparasit. Kutu dan sengkenit adalah ektoparasit yang paling prevalens pada ruminan kecil dalam kajian ini dengan sebanyak 43.64% dan 22.98% haiwan dijangkiti olehnya. Keupayaan biotik tinggi yang dimiliki oleh kutu adalah antara salah satu faktor yang menjadikan ia sebagai ektoparasit yang paling prevalens pada ruminan kecil. Tiada hubungan yang signifikan di antara prevalens ektoparasitisme dan spesies ruminan kecil (χ^2^ = 1.12, *p* = 0.293). Walau bagaimanapun, terdapat variasi signifikan di antara kawasan di mana sampel diambil (χ^2^ = 30.25, *p* = 0.002) dan sistem pengurusan ladang untuk keduadua spesies. Tambahan pula, pinjal dan tungau adalah kurang prevalens pada ruminan kecil di Kelantan. Hasil kajian ini memberikan dasar data epidemiologi pada prevalens ektoparasitisme pada ruminan kecil. Maklumat ini juga penting sebagai pencegahan dan kawalan dalam meningkatkan produktiviti ruminan di Kelantan.

**Highlights**

- 60% of the sampled animals were infested with at least one species of the ectoparasites.
- Lice and ticks were the most prevalent ectoparasites on small ruminant, which were 43.64% and 22.98% respectively.
- The prevalence of ectoparasites on small ruminant is significantly higher at inland region.

## INTRODUCTION

Kelantan is a chiefly agrarian state located in the northeast region of Peninsular Malaysia. It has ambient temperatures ranging from 24°C to 33°C (WorldData.info 2019) and a distinct October to March monsoon season. Small-scale backyard and small-holder ruminant production are common practices in Kelantan as are in other regions of Malaysia (Mohamed 2007). Farmers rear small ruminants, sheep and goats for their high fertility and short generation interval (in comparison to cattle). These traits are minimally affected by harsh environmental conditions and hence their production serve as source of secondary income and insurance for locals (Olafadehan *et al.* 2014).

Even though small ruminants accounts for 5% to 10% of Malaysian National Gross Domestic Products (GDP), their contribution to food production and national meat and milk self-sufficiency as well as supporting rural income are far below desired levels (Mohamed 2007). The major constraints to small ruminant production in Malaysia are diseases, limited feed resources and poor management systems. Ectoparasitism being a disease entity caused by parasitic infestation is able to cause overall deterioration of small ruminants’ production and reproduction that eventually lead to serious economic losses to the small-holder farmers and the country as a whole (Shanmugavelu 2014).

Heavy infestation of ectoparasites such as lice, ticks, mites and fleas cause lower skin and hide quality, poor body condition along with reduction in milk and meat production (Abebe *et al.* 2011). Medical management of the diseases transmitted by these arthropods are accounted for economic losses to the farmers (Seyoum *et al.* 2015).

Despite of the economic and animal health importance of these ectoparasites, there is a lack of information on their prevalence and spatial distribution in small ruminants in the East Coast economic region of Malaysia. Therefore, the objectives of this present study are to determine the prevalence of ectoparasites in small ruminants in Kelantan and identify the risk factors associated with the ectoparasitism.

## MATERIALS AND METHODS

### Study Area

Field work was conducted in 10 districts of Kelantan, Malaysia within latitude 6°07’31.43” N and longitude 102°14’17.06” E. Sampling was conducted between April to September 2017 corresponding to the dry season. Farms were randomly selected from each of the 10 districts irrespective of their farm management systems *viz*. intensive, semi-intensive and extensive. Farms were identified and randomly selected from the Department of Veterinary Services, Kelantan database.

### Sample Size Determination

Number of animals required to measure the prevalence of ectoparasites was calculated based on 50% expected prevalence (used to maximise sampling) at 95% confidence level and 5% desired absolute precision (Thrusfield 2005). Hence, the minimum number of animals utilised for the study was 384 (192 of sheep and 192 of goats).

### Sampling Methods

Parameters such as age, gender, body score and farm management practices were recorded prior to the physical examination and sample collection. Survey forms (questionnaires) were distributed to farmers to obtain additional background information of the farm. The selected sheep and goats were physically examined for the presence of ectoparasites and lesions. Physical examination of the integumentary system was performed by multiple fleece parting in the direction opposite to hair normally rests as well as by visual inspection. Ectoparasites such as lice, ticks and fleas were collected manually (by hand or forceps) and preserved in separate vials containing 70% ethanol (Urquhart *et al.* 1996). Skin scrapings for mites were obtained from sheep and goats with mange-associated skin lesions (such as crustiness) and processed according to the method described by Cole (1986).

### Ectoparasites Identification

Collected samples were examined under stereomicroscope for lice, tick and fleas through their morphological features. The identification up to genus level was performed by referring to the identification key in the guidelines given by Walker *et al*. (2003) for ticks and Wall and Shearer (2008) for lice, fleas and mites.

## DATA MANAGEMENT AND ANALYSIS

Data management was conducted by using Microsoft Excel^®^ and all recorded data were analysed using Statistical Package for Social Science (SPSS^®^) version 20. Descriptive statistics, percentages and 95% confidence interval were used to summarise the proportion of infested and non-infested animals. The association with different risk factors on the prevalence and distribution of ectoparasites were analysed using Chi-square test. The differences were considered as significant when at 95% confidence intervals while statistical significance was set at *p* < 0.05.

## RESULTS AND DISCUSSION

The prevalence of ectoparasitism in small ruminants was significantly associated with the region where the animals were sampled from (χ^2^ = 30.25, *p* = 0.002158). In the coastal region of Kelantan, the prevalence of ectoparasitism was 50.71%; while in the inland region the prevalence of ectoparasitism was 73.71% (Table 1). This study observed a statistically significant relationship in prevalence between different regions of the state (*p* < 0.05). Ectoparasitism in sheep and goats was higher in the inland areas of Kelantan with a prevalence of 73.71% in comparison to coastal areas (50.71%). The high prevalence of ectoparasitism in the inland regions of Kelantan is associated with the presence of conserved forested areas which provides favourable climatic and environmental conditions for the thriving of ectoparasites. Geographical factors are known to affect the distribution of many animal species and thus ectoparasites (Watanabe *et al.* 2016). Other researchers have also presented similar results for inland and coastal regions in different countries (Ghosh *et al.* 2007; Bermudez & Miranda 2011; Tamerat *et al.* 2016).

There was a statistically significant association between the prevalence of caprine ectoparasitism and farm management system (χ^2^ = 6.06, *p* = 0.048). Higher prevalence caprine ectoparasitism was observed in farms practicing semi-intensive management system (73.33%) followed by extensive (67.39%) and intensive (59.69%) management systems (Table 2). There was also a statistically significant association between prevalence of ovine ectoparasitism and farm management system (χ^2^ = 51.12, *p* < 0.0001). Higher prevalence of ectoparasitism in sheep was observed in farms practicing semi-intensive management system (78.42%) while farms practicing the intensive system had 30.68% prevalence (Table 2). Extensive farm management was not practiced for sheep in the farms studied in Kelantan. The current study revealed that farm management practices has statistically significant relationship with the prevalence of ectoparasitism in small ruminants (*p* < 0.05) in Kelantan. Extensive and semi-intensive management has high prevalence of ectoparasites due to higher risk of exposure to ectoparasites during grazing (Wall & Shearer 2008). In semi-intensive management practices exposed animals easily spread the ectoparasites to other animals in the farm on return via contact (Diaz 2006).

**Figure 1:**
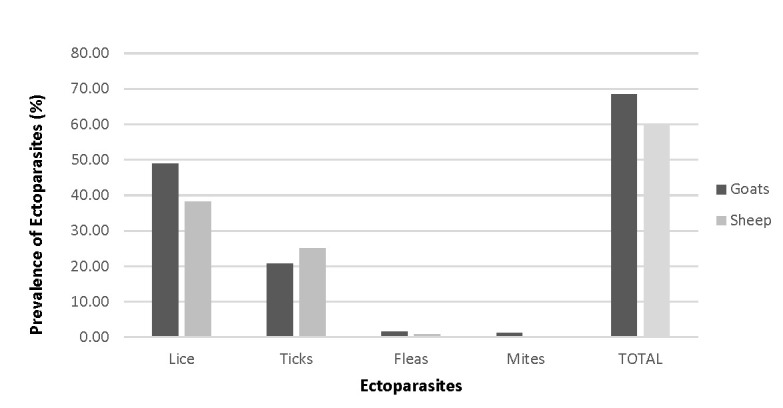
The overall prevalence of ectoparasitism on sheep and goats in Kelantan.

In this study, the overall prevalence of small ruminant ectoparasitism was 63.20% (292/462). The highest ectoparasite was lice, which were 43.64% discovered in the small ruminant. The optimum temperature and humidity in the study area attributed to the reproduction of lice on these small ruminants. This study conducted on a tropical environment. Tropical climates promote a higher number of parasitic load in small ruminants (Azfan 2018). Subsequently, with tick, which was 22.98% found in this study. Ectoparasitism prevalence in sheep was found to be 59.91% (136/227) while in goats 64.68% (156/235). There was a statistically significant difference in ectoparasite prevalence between sheep and goats (χ^2^ = 4.36, *p* = 0.038). The prevalence of ectoparasites identified on goats were lice (48.94%) followed by ticks (20.85%), fleas (1.70%) and mites (1.28%). Similar findings were observed for sheep with lice being the dominant ectoparasite (38.33%), followed by ticks (25.11%) and fleas (0.88%) (Fig. 1). The prevalence of caprine ectoparasitism of the present study differs from what had been reported by Khadijah *et al.* (2014), where 80% of caprine ectoparasitism were reported in Kuala Terengganu, Peninsular Malaysia. The prevalence of ectoparasites infestation were higher in Terengganu because the smaller number of animals was sampled and the area that had been covered also limited. The variation of ectoparasitism prevalence in different study areas also could be due to differences in the management, agro-climate, seasons, health care and ectoparasite control practices in the respective study areas (Tamerat *et al.* 2016). It is evident that the prevalence of small ruminant ectoparasitism in this study is high in Kelantan, thus indicating a chronic underreporting of the condition in the state. This study identified an overall 62.99% prevalence of ectoparasites infestation; out of which 59.91% was the prevalence of ovine ectoparasitism and 64.68% for caprine ectoparasitism. This finding is in line with that of Jarso *et al.* (2014) who reported ectoparasitism prevalence of 10.3% for sheep and 16.5% and goats in the Kombolcha region of North East Ethiopia. In a similar study in Ethiopia reported an ectoparasitism prevalence of 42.2% and 51.4% for sheep and goats, respectively (Tamerat & Zeryehun 2015).

**Figure 2:**
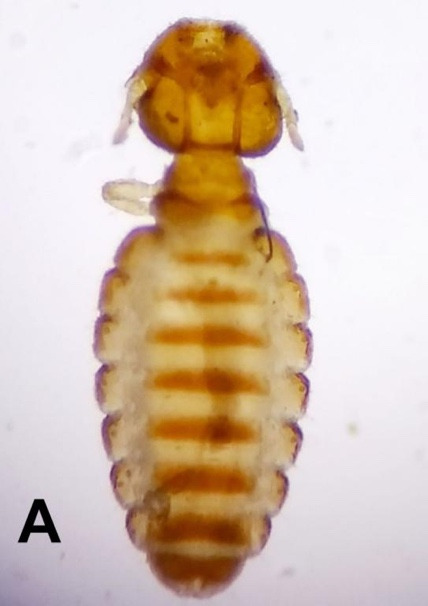

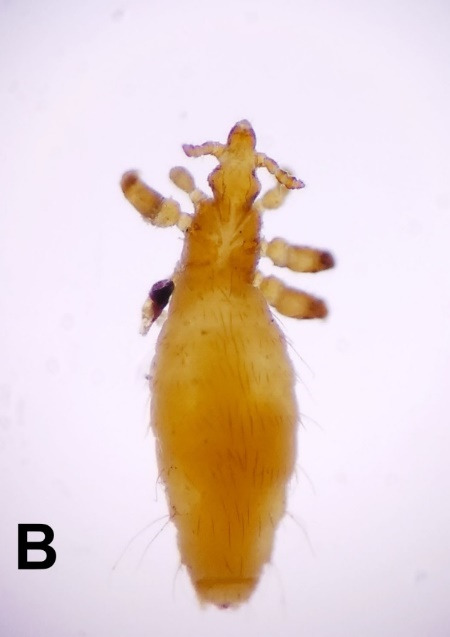

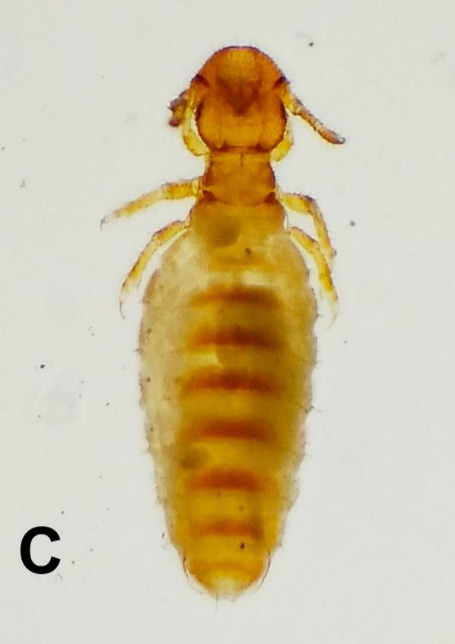
Photographs of morphologically identified three species of lice on small ruminants in Kelantan. (A) *Bovicola caprae* on goat; (B) *Linognathus africanus* on goat; (C) *Bovicola ovis* on sheep.

*Bovicola caprae* and *Linognathus africanus* were found on the goat meanwhile only *Bovicola ovis* was found on the sheep (Fig. 2). Lice species that were found on individual animals studied, where chewing lice (*Bovicola* sp.) were widely distributed on the animal’s body than the sucking lice (*Lignonathus* sp.). This study revealed that lice and ticks are common ectoparasites on small ruminants in Kelantan. Yakhchali and Hosseine (2006) reported that lice and ticks are common ectoparasites on small ruminant in Urmia suburb, Iran. Moreover, Mbuh *et al.* (2008) also reported only tick and lice that were common ectoparasites on small ruminants in Bokova, a rural area of Buea Sub Division, Cameroon. Among the ectoparasites detected, lice were found as the most prevalent in both sheep and goat, which comprised 43.72% of the total prevalence. This result in concordance with previous studies that reported lice as the most prevalent ectoparasite species in small ruminants (Eticha *et al.* 2017; Getaneh *et al.* 2016; Seyoum *et al.* 2015). The high prevalence of lice might due to the high biotic potential of lice population in the host (Bates 2012). As the entire lifecycle of lice is completed within one host, population of lice can be rapidly established once they come into contact with a suitable host. Spreading of lice to other animals within same herd is not uncommon as the transmission of lice can be achieved through close proximity contact (Hart 1994).

**Figure 3:**
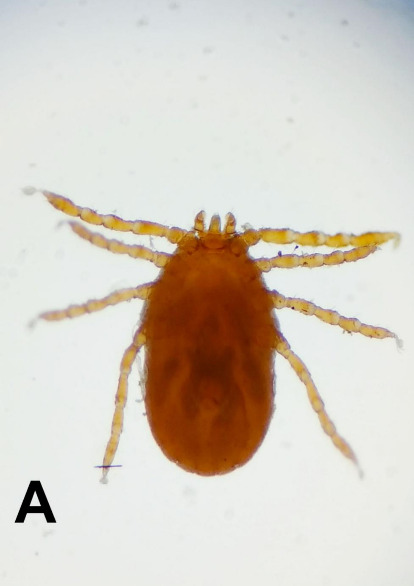

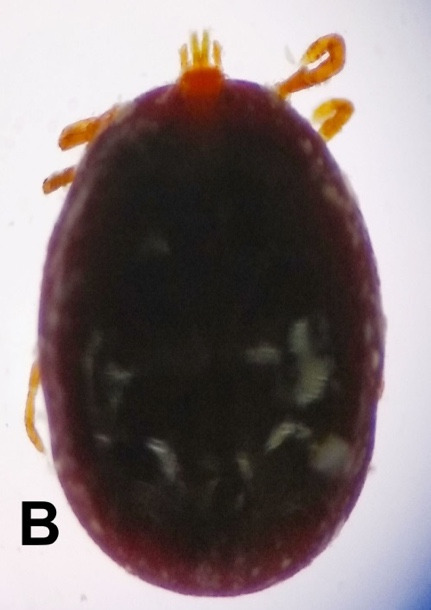

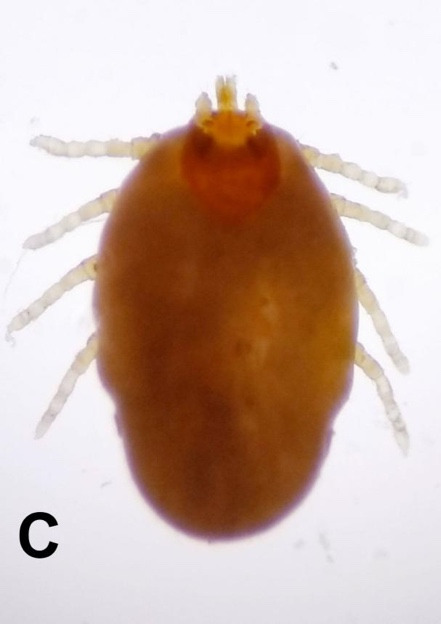
Genus of tick identified from small ruminants in Kelantan. (A) *Haemaphysalis* spp.; (B) *Boophilus* spp.; (C) *Rhipicephalus* spp.

Result from this study revealed that tick’s prevalence was higher in sheep compared to goat. This was in agreement with Fentahun *et al.* (2012) and Yacob *et al.* (2008), who also reported a high prevalence of ticks in sheep than in goats. The variation in tick prevalence on different species is be attributed to a variety of factors such as behavioural characteristics of different species of animals, awareness/education of the farmers and farm management practices (Ghosh *et al.* 2007). Tick collected from sheep and goats in this study belonged to four genera which are *Haemaphysalis*, *Boophilus*, *Rhipicephalus* and *Amblyomma.* Three tick genera, *Haemaphysalis*, *Boophilus* and *Rhipicephalus* were found to be common on both sheep and goats while the genus *Amblyomma* was only present on sheep (Fig. 3). The study by Irshad *et al.* (2010) revealed that *Rhipicephalus*, *Haemaphysalis*, *Ixodes* and *Ambylomma* were found in sheep and goats at two different livestock farms located in the Potohar region, namely Barani Livestock Production Research Institute (BLPRI) Kherimurat, District Attock and National Agricultural Research centre (NARC), Islamabad, Pakistan. Another study by Jafer *et al.* (2017) reported that *Boophilus*, *Rhipicephalus*, *Amblyomma* and *Hyalomma* were found on small ruminants in and around Dire Dawa, eastern Ethiopia. On the other hand, three genera of ticks *viz*. *Boophilus*, *Amblyomma* and *Rhipicephalus* were identified both on sheep and goats by Sertse and Wossene (2007) in eastern part of Amhara region, northeast Ethiopia and by Adang *et al.* (2015) in the northeast of Nigeria.

**Figure 4:**
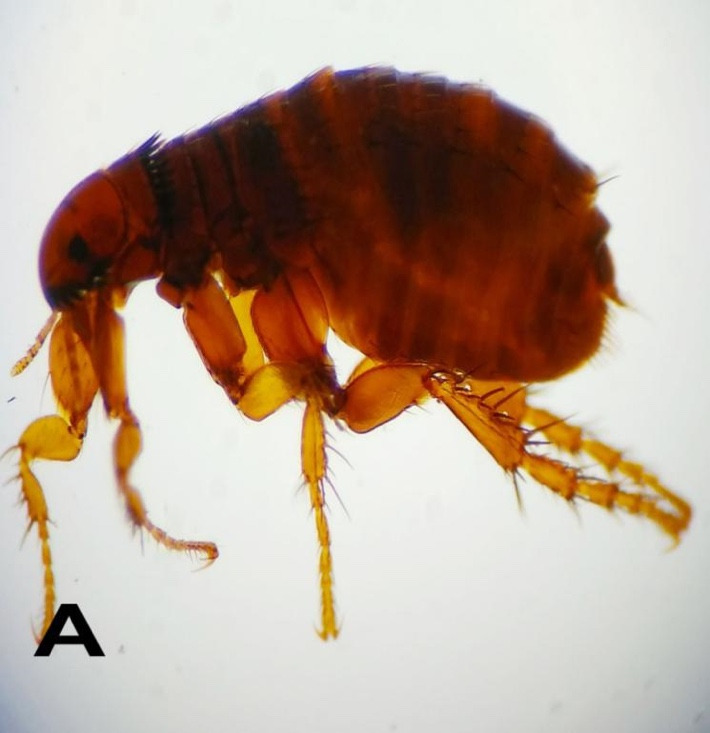

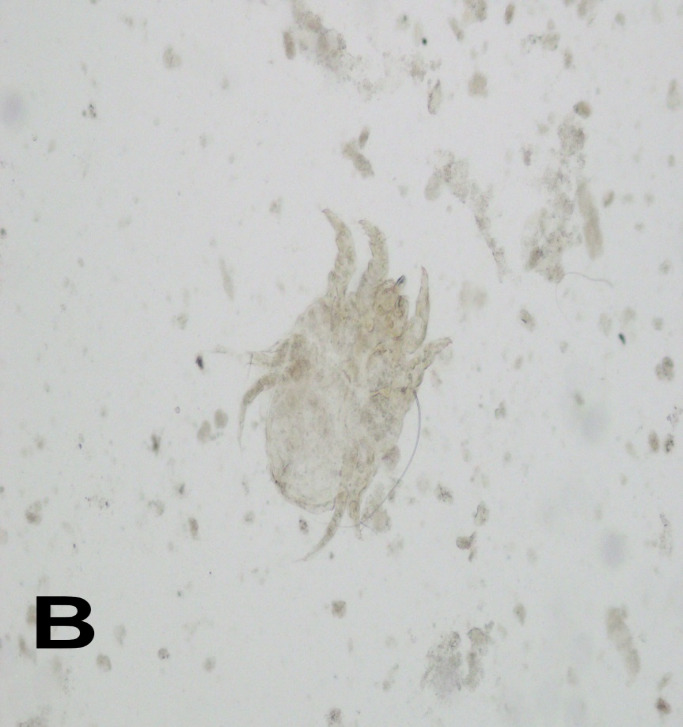
Other species of ectoparasites identified on small ruminants in Kelantan. (A) *Ctenocephalides* spp.; (B) *Sarcoptes* spp.

The flea, *Ctenocephalides* sp. was found on both sheep and goats reared in farms with poor management practice (Fig. 4). The prevalence rate for flea infestation in this study area – Kelantan, was 1.30%. This study showed lower prevalence compared to the study done by other researchers in Nigeria, northwest Ethiopia, southern Ethiopia, northeast Ethiopia and in Tanzania (Kusiluka *et al.* 1995; Sertse & Wossene 2007; Yacob *et al.* 2008; Fentahun *et al.* 2012; Adang *et al.* 2015; Seyoum *et al.* 2015). This is probably associated with the lower humidity on the study area during the dry season with the average of relative humidity of 60%, when > 70% of relative humidity is usually required for survival at the high temperature (Soulsby 1968).

Mange mites, *Sarcoptes* sp. were found only on goats in few farms (Fig. 4). The mange mite *Sarcoptes* was found to be of lesser prevalence (1.28%) than other ectoparasites in goats in Kelantan. This result is lower compared to Dorny *et al.* (1994) who reported a prevalence of mites (88%) on goats in Selangor and Perak. Kelantan also has a lower prevalence of mange mites compared to reports from other countries, 20% (UK) by Lusat *et al.* (2009), 52% (north-western Kenya) by Wafula *et al.* (2008) and 100% of caprine flock (north-eastern Italy) by Menzano *et al.* (2007). These differences in prevalence might be due to difference in animal population which causes unfavourable condition for the transmission of mites between animal and result in lower level of mite infestation (Tesfaheywet & Simeon 2016). Besides, *Sarcoptes* sp. was the only one genus that found in this study. This is similar findings from the previous study done by León-Vizcaíno *et al.* (1999) and Gabaj *et al.* (1992), who reported that *Sarcoptes* was the common mites found on goats.

## CONCLUSION

In conclusion, this present study revealed that high prevalence of small ruminant ectoparasitism could be the major causes of sheep and goat production constraints and quality deterioration. Ectoparasitism in sheep and goats was higher in the inland areas compared to the coastal area. Our result also showed that semi-intensive and extensive management system have the highest ectoparasitism rate. Hence, it would be valuable to implement effective extension system for the management and more programs that could lift up community awareness based on the risk factors as mentioned in this study. This particularly dedicated on farm management, effect of ectoparasites, and strategic practice of control measures with full cooperation from farmers and authorities (Veterinary department) in the districts area. More studies on ectoparasitism needs to be conducted in this region and Peninsular Malaysia to provide information on prevention and control measure in order to farmers’ income and improve agriculture industry.

## Figures and Tables

**Table 1:** The overall prevalence of ectoparasitism in different regions in Kelantan.

Regions	District	Infested animal (n)	Total (N)	Prevalence (%)	Average prevalence (%)
Coastal	Kota Bharu	32	68	47.06	50.71
	Bachok	29	63	46.03	
	Pasir Putih	13	40	32.50	
	Tumpat	33	40	82.50	

Inland	Pasir Mas	38	47	80.85	73.71
	Jeli	25	30	83.33	
	Tanah Merah	20	36	55.56	
	Kuala Krai	19	38	50.00	
	Gua Musang	36	47	76.60	
	Machang	47	53	88.68	

**Table 2:** Prevalence of sheep and goats ectoparasitism according to farm management systems in Kelantan.

Small ruminants	Intensive	Semi-intensive	Extensive
Sheep	30.68%	78.42%	–
Goats	59.69%	73.33%	67.39%
